# gsGator: an integrated web platform for cross-species gene set analysis

**DOI:** 10.1186/1471-2105-15-13

**Published:** 2014-01-14

**Authors:** Hyunjung Kang, Ikjung Choi, Sooyoung Cho, Daeun Ryu, Sanghyuk Lee, Wankyu Kim

**Affiliations:** 1 Ewha Global Top5 Research Program, Ewha Womans University, 52 Ewhayeodae-gil, Seodaemun-gu, Seoul 120-750, Korea; 2 Ewha Research Center for Systems Biology (ERCSB), Ewha Womans University, 52 Ewhayeodae-gil, Seodaemun-gu, Seoul 120-750, Korea

**Keywords:** Gene set analysis, Cross-species GSA, Interactive GSA, Gene function, Omics

## Abstract

**Background:**

Gene set analysis (GSA) is useful in deducing biological significance of gene lists using a priori defined gene sets such as gene ontology (GO) or pathways. Phenotypic annotation is sparse for human genes, but is far more abundant for other model organisms such as mouse, fly, and worm. Often, GSA needs to be done highly interactively by combining or modifying gene lists or inspecting gene-gene interactions in a molecular network.

**Description:**

We developed *gsGator*, a web-based platform for functional interpretation of gene sets with useful features such as cross-species GSA, simultaneous analysis of multiple gene sets, and a fully integrated network viewer for visualizing both GSA results and molecular networks. An extensive set of gene annotation information is amassed including GO & pathways, genomic annotations, protein-protein interaction, transcription factor-target (TF-target), miRNA targeting, and phenotype information for various model organisms. By combining the functionalities of *Set Creator, Set Operator and Network Navigator*, user can perform highly flexible and interactive GSA by creating a new gene list by any combination of existing gene sets (intersection, union and difference) or expanding genes interactively along the molecular networks such as protein-protein interaction and TF-target. We also demonstrate the utility of our interactive and cross-species GSA implemented in gsGator by several usage examples for interpreting genome-wide association study (GWAS) results. gsGator is freely available at http://gsGator.ewha.ac.kr.

**Conclusions:**

Interactive and cross-species GSA in gsGator greatly extends the scope and utility of GSA, leading to novel insights via conserved functional gene modules across different species.

## Background

High-throughput experiments such as microarray, next generation sequencing and mass spectrometry-based proteomics, provide genome-scale molecular profile data in an unbiased manner. Gaining biological insights on underlying mechanisms, however, requires interpretation of several hundreds or even thousands of candidate genes. Gene set analysis (GSA) has been highly successful to interpret the result from high throughput experiments and a number of GSA tools have been developed such as DAVID [1], GeneCodis3 [2], WebGestalt2 [3], g:Profiler [4], GARNET [5], ToppCluster [6], just to name a few.

Still, current progress on functional genomics annotation is far from complete. Particularly, phenotypic annotation is sparse for human genes, but is far more abundant for other model organisms such as mouse [7], fly [8], and worm [9]. However, cross-species GSA has not been extensively used to interpret human gene lists. Interpretation of omics experiments usually is done in a highly interactive manner. For example, the gene list from the same experiment (*e.g.* microarray) needs to be analyzed multiple times using different stringency criteria. Several gene sets from related experiments need to be combined for another GSA by operating set union, intersection or difference. Analysis of gene lists in the context of molecular networks may lead to novel mechanistic insights. For example, a gene list can be trimmed by taking only genes directly connected to each other or, alternatively, expanded to include network neighbors in different types of molecular network. Flexible combination of such interactive features is highly desirable for any web-based GSA tool, which greatly increases its sensitivity and interpretability.

While the utility of interactive and cross-species GSA is evident, few tools support such functionality in a single, unified environment (Table 1). Here, we developed a web-based tool, gsGator with many useful features such as cross-species GSA and a network viewer. The whole analysis is virtually automated with a convenient drag-and-drop interface. A broad range of gene annotations are collected for seven common model organisms of human, mouse, fly, worm, yeast, *Arabidopsis* and *E. coli*. Ample information on phenotypes and functions is available for these model organisms, and the full list of annotation types and their statistics is available on-line.

**Table 1:** Comparison of the supported features in GSA tools

**Tool name**	**Annotation types**					**Multiple GSA**	**Cross-species GSA**	**Set operation (-,∩,∪)**	**Network analysis & visualization**	**No. species**	**Ref.**
	**GO & pathway**	**Genomic annotation**	**Molecular network**	**Phenotype**	**miRNA target**						
gsGator	O	O	O	O	O	O	O	O	O	7 species	
DAVID	O	O	O	O		O		O		All species	[1]
g:Profiler	O		O	O	O	O	O			85 species	[4]
Algal Functional Annotation Tool	O	O								2 fungal species	[10]
GeneTerm Linker	O	O								H. sapiens yeast	[11]
KOBAS 2.0	O			O			O			1327 species	[12]
ADGO 2.0	O			O						8 species	[13]
ToppCluster	O	O	O	O	O	O				H. sapiens	[6]
PhenoFam		O								48 species	[14]
agriGO	O									38 species	[15]
Lists2Networks	O	O	O	O	O	O		O	O	Mammals	[16]
GeneCodis3	O	O				O		O		16 species	[2]
FuncAssociate (ver 2.0)	O									37 species	[17]
GeneWeaver	O			O	O	O	O	O		9 species	[18]

## Construction and content

**Figure 1:**
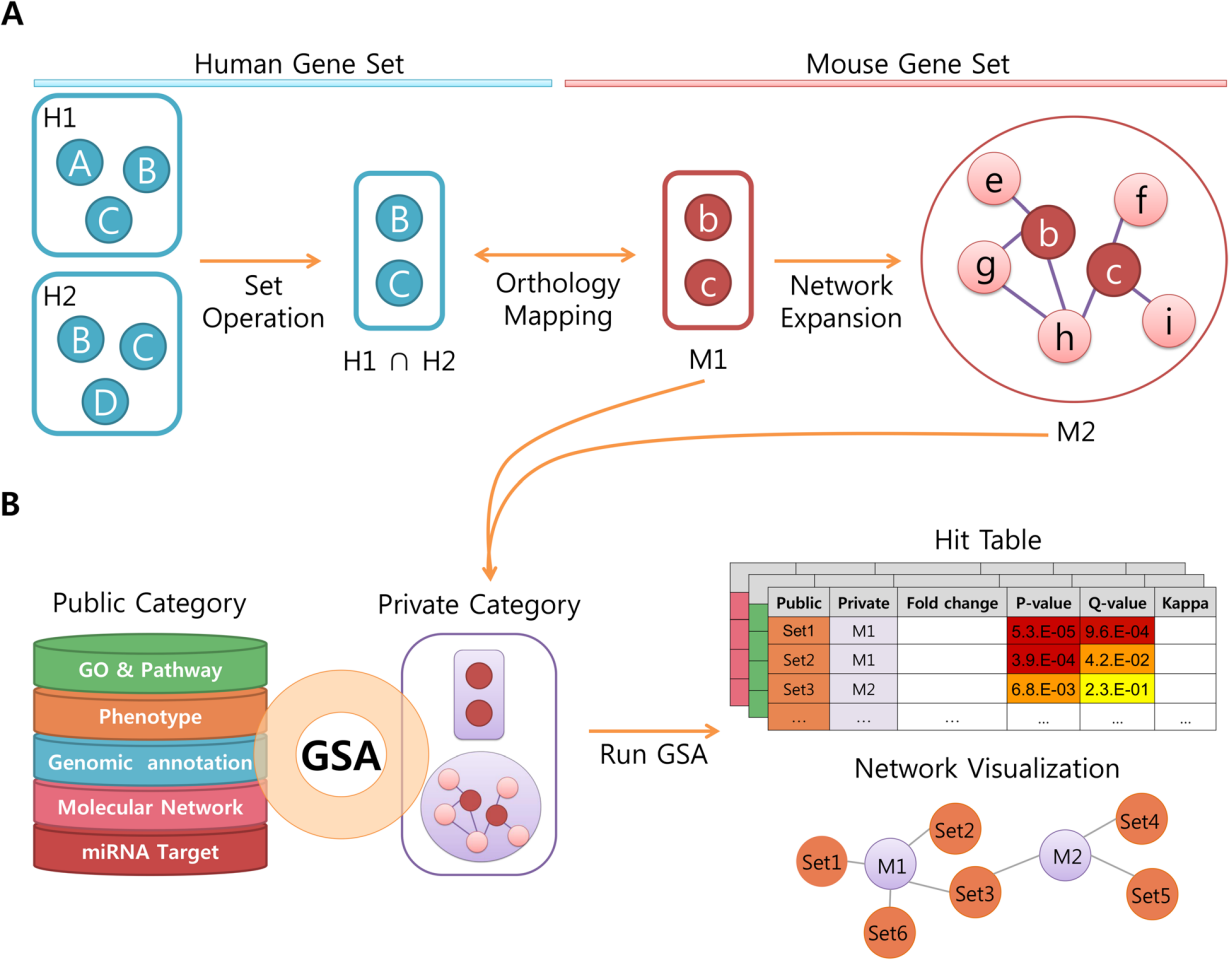
**A typical example of interactive and cross-species GSA in gsGator. A**. Preparation of input gene set using set operation, orthology mapping, and network expansion tools. Two human gene sets (H1, H2) are combined to generate H1∩H2 by intersection operation. An orthologous set of M1 is generated by orthology mapping to mouse genes from H1∩H2. The M1 set is expanded via a molecular network, resulting in M2. **B**. GSA is performed between predefined gene sets in the public category and M1, M2 in the private category. The GSA result is shown as a table or a network among gene sets, where significantly associated gene sets are connected to each other.

The gsGator consists of four main modules of set creator, set operator, set analyzer and network navigator. Following is the brief introduction of the main features in each module and its user interface. We chose NCBI Entrez Gene ID as unique gene identifier throughout the whole system. The pre-compiled gene sets are listed under ‘public’ category and the user-input gene sets under ‘private’ category. In gsGator, GSA is always performed between the private and the public annotations. A typical analytic procedure in gsGator is shown in Figure 1.

**Set Creator** lets user define new gene sets *de novo*, where the input gene/protein ids are automatically converted to Entrez Gene ID (Additional file 1: Table S1). Gene lists can be directly put in the input box or uploaded as a file. Set creator is equipped with orthology mapping tool, where any gene set can be converted to its orthologous set for multiple model organisms in a single step. For each model organism, a separate gene set is created e.g. ‘example_set_yeast’, ‘example_set_mouse’, etc. for a set named 'example_set'. Currently, orthology mapping is done using the information from InParanoid database [19]. All the user-created gene sets are deposited in the private category.

**Set Analyzer** lets user perform gene set analysis (GSA) using a convenient drag-and-drop user interface. Simultaneous GSA of multiple gene sets is allowed. First, user selects the target species, where human (*Homo sapiens*) is set as default. Once a species is selected, only the relevant gene sets to the species are listed under the category tree for both public and private section. User can select the target gene sets from both public and private category by drag-and-drop using mouse. Because GSA is performed between the selected private and public categories, at least one private and one public category should be selected under the input area. The significance of GSA is calculated using hypergeometric test and kappa statistics, where multiple test correction is applied by Benjamini-Hochberg method. The result of GSA is presented in a table with its statistical significances (p-value and q-value) presented as heatmap. The GSA result can be also visualized as a network for an intuitive inspection of the results.

**Set Operator** is for creating a new gene set by the union, intersection or difference of two preexisting gene sets. The resulting gene list is directed to *set creator* for generating a new gene set.

**Network navigator** allows user to explore molecular networks such as protein-protein interaction (PPI), TF-target and miRNA-target relations. Starting with a particular gene set as seed, the user can expand genes along the molecular networks in an interactive fashion. Selecting a node and right-click on the mouse triggers a pop-up menu to choose the type of network for expansion. Once the modification of network is complete, the remaining nodes (genes) can be exported to *set creator* to generate a new gene set. Combined use of *set operator* and *network navigator* allows user to create a new gene set in a highly flexible and interactive manner using any preexisting gene sets.

### Utility and discussion

**Figure 2:**
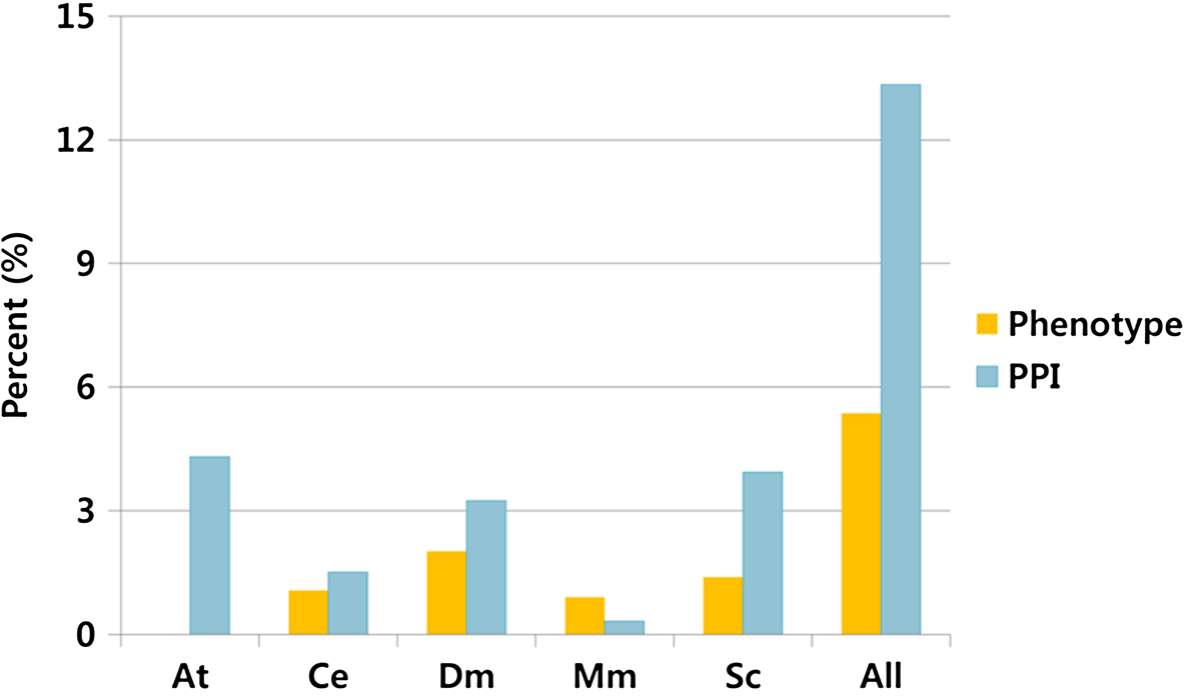
**The gain of annotation coverage for human genes by orthology mapping for phenotype (orange) and protein-protein interaction network (blue) in gsGator.** (At: *A. thaliana*, Ce: *C. elegans*, Dm: *D. melanogaster*, Mm: *M. musculus*, Sc: *S. cerevisiae*, All: all the 5 model organisms).

According to our survey on the datasets in gsGator, the fraction of human genes with any phenotypic annotation is only 40.9%, most of which are genetic diseases (Table 2). Because a single gene is frequently associated with many phenotypes, this number of annotation coverage should be overestimated in reality. Gene annotation from model organisms is a rich source for inferring the function of human genes. By taking advantage of phenotype and protein-protein interaction network of orthologous genes from other model organisms, the coverage for human genes increases by 5.4% and 13.3%, respectively (Figure 2). This gain of phenotypic information is likely to increase for a while, because the rate of phenotypic characterization is likely to be much faster for model organisms than human. Similarly, 12% of additional coverage is gained for protein-protein interaction (PPI) network. Although gene function and network structure may have diverged significantly between human and model organisms, functional gene modules are often unexpectedly well conserved by deep homology [20].

**Table 2:** The coverage of gene functional annotation and molecular networks by major annotation DBs

		**Hs**	**At**	**Ce**	**Dm**	**Mm**	**Ec**	**Sc**	**Total gain**
	**Total number of genes**	42,059	33,584	45,727	22,930	57,992	4,497	6,353	
**All annotation**	**Number of annotated genes (% Coverage)**	34,710 (82.5%)	27,274 (81.2%)	18,734 (41.0%)	20,106 (87.7%)	48,128 (83.0%)	2,531 (56.3%)	6,303 (99.2%)	
**Phenotype Annotation**	**Genes with phenotype annotation (% Coverage)**	17,198 (40.9%)	0	6,513 (14.2%)	16,665 (72.7%)	9,966 (17.2%)	0	5,620 (88.5%)	
	**The gain of inferred annotation by orthology for human genes (% Coverage)**		0	447 (1.1%)	848 (2%)	377 (0.9%)	0	583 (1.4%)	2,255 (5.4%)
**PPI**	**Genes connected by PPI network (% Coverage)**	9,621 (22.9%)	6,911 (20,6%)	2,681 (5.9%)	5,622 (24.5%)	1,588 (2.7%)	47 (1.0%)	5,479 (86.2%)	
	**The gain of PPI network coverage by ortholgy (% Coverage)**		1,814 (4.3%)	634 (1.5%)	1,365 (3.2%)	138 (0.3%)	0	1,658 (3.9%)	5,609 (13.3%)

**Figure 3:**
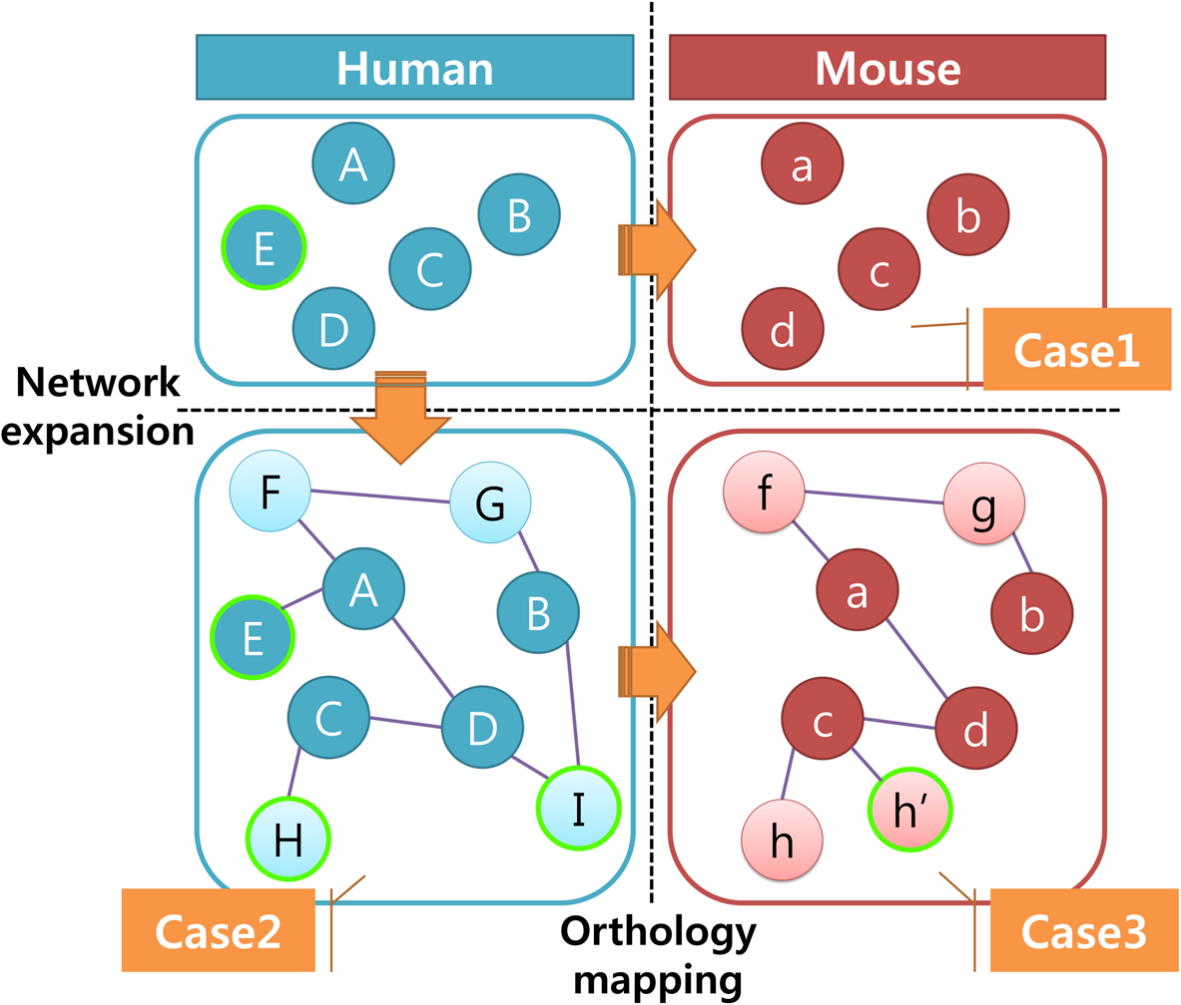
**A schematic diagram of preparing the list of input genes in the three case examples.** In case 1, the input human genes are simply mapped to mouse genes by orthology. In case 2, the human genes are expanded by molecular network e.g. protein-protein interaction (PPI). In case 3, the input genes are first, network expanded and then mapped to mouse genes by orthology. Some of the human genes may have no orthologous (E and I) or multiple orthologous genes (H → h, h’) in mouse.

In order to test the utility of gsGator, we focus on mouse phenotypic annotation because of the paucity of human counterpart and thus, the benefit of interactive and cross-species GSA can be more evident. Here, we took three example cases, where GSA was performed using GWAS hit genes as input. Typically, GWAS identifies genetic variations associated to certain phenotypes, resulting in a small number of associated genes as hits. The paucity of human phenotypic annotation makes it difficult to cross-confirm GWAS results. Frequently, GWAS hit genes do not belong to the same pathway or are not directly connected to each other in molecular networks, making it hard to gain mechanistic insight. The number of GWAS hit genes is often too small to get enough statistical significance by conventional GSA. In the three case examples below, the GSA results by conventional GSA for human phenotypic annotation is compared with those by GSA using the features of 1) cross-species GSA, 2) Network expansion of input genes and 3) Network expansion + cross-species GSA. The procedure of preparing the input genes is illustrated in Figure 3. The details of the analytic procedures are also available in the Additional file 2 including all the input gene sets and the step-by-step screenshots of gsGator web interface.

### Case example 1: Cross-species GSA

In the first case, five genes from a genome-wide association (GWA) study for eye color were used as an input gene list [21]. Conventional GSA resulted in no significant hits for human phenotypic annotation [22,23]. However, a simple cross-species GSA identified many related annotations using mouse phenotypic annotations such as *decreased eye pigmentation* (q-value = 2.5e-6) and *diluted coat color* (q-value = 2.8e-5) (Table 3).

**Table 3:** The GSA result by a simple cross-species GSA (case example 1)

**Rank**	**Set size**	**Overlap**	**P-value**	**Q-value**	**Mouse phenotype**
**1**	**22**	**3**	**3.3E-10**	**2.5.E-06**	**Decreased eye pigmentation**
**2**	**30**	**3**	**8.7E-10**	**3.3.E-06**	**Abnormal eye pigmentation**
**3**	**51**	**3**	**4.5E-09**	**1.1.E-05**	**Abnormal skin pigmentation**
**4**	**76**	**3**	**1.5E-08**	**2.8.E-05**	**Diluted coat color**
**5**	**87**	**3**	**2.3E-08**	**3.4.E-05**	**Abnormal coat/hair pigmentation**
**6**	**5**	**2**	**5.2E-08**	**5.6.E-05**	**Absent eye pigmentation**
**7**	**5**	**2**	**5.2E-08**	**5.6.E-05**	**Ocular albinism**
**8**	**7**	**2**	**1.1E-07**	**1.0.E-04**	**Variegated coat color**
**9**	**8**	**2**	**1.5E-07**	**1.1.E-04**	**Abnormal choroid melaningranule morphology**
**10**	**8**	**2**	**1.5E-07**	**1.1.E-04**	**Mottled coat**
**11**	**9**	**2**	**1.9E-07**	**1.3.E-04**	**Abnormal melanogenesis**
**12**	**18**	**2**	**7.9E-07**	**5.0.E-04**	**Yellow coat color**
**13**	**24**	**2**	**1.4E-06**	**8.3.E-04**	**Abnormal melanosome morphology**
**14**	**27**	**2**	**1.8E-06**	**9.8.E-04**	**Irregular coat pigmentation**
**15**	**31**	**2**	**2.4E-06**	**1.2.E-03**	**Hypopigmentation**
16	87	2	1.9E-05	9.1.E-03	Prenatal lethality
17	975	3	3.2E-05	1.4.E-02	Decreased body size
18	125	2	4.0E-05	1.7.E-02	Infertility
19	237	2	1.4E-04	5.4.E-02	Increased susceptibility to bacterial infection
20	365	2	3.4E-04	9.1.E-02	Complete prenatal lethality
21	519	2	6.8E-04	1.1.E-01	Male infertility
22	574	2	8.3E-04	1.3.E-01	Complete postnatal lethality
23	1258	2	3.9E-03	2.9.E-01	Premature death
24	1642	2	6.5E-03	3.9.E-01	No abnormal phenotype detected

The related annotations to eye color is indicated in bold type.

### Case example 2: Network expansion

The second case takes a bit more elaborate approach, where the results of two GWA studies for adiposity [24,25] were combined by the union (seven genes) of two hit gene lists (three and four genes respectively). Neither conventional nor simple cross-species GSA resulted in any significant GSA hits for phenotypic annotation. In the *network navigator*, we observed that none of the seven input genes were connected to each other by PPI network. By expanding each input gene via PPI network, a larger input set of 104 genes was created, where four of the initial seven genes were indirectly connected by the expanded network neighbors as intermediate. GSA using the network-expanded 104 genes as input provided even rich phenotypic interpretations with many GSA hits on energy metabolism including *insulin resistance* (q-value = 5.0e-3), *body fat* (q-value = 9.6e-3), and *glucose tolerance* (q-value = 4.3e-2) (Table 4). This example demonstrates that network-expanded GSA allows even more sensitive and extensive interpretation of gene lists with improved statistical significance.

**Table 4:** The GSA result by a network-expanded GSA (case example 2)

**Rank**	**Set size**	**Overlap**	**P-value**	**Q-value**	**Human phenotype**
**1**	**44**	**4**	**9.2.E-06**	**5.0.E-03**	**Insulin**
2	3	2	2.7.E-05	7.2.E-03	Polycystic ovarian syndrome
**3**	**4**	**2**	**5.3.E-05**	**9.6.E-03**	**Body fat**
**4**	**5**	**2**	**8.8.E-05**	**1.2.E-02**	**Insulin resistance**
**5**	**10**	**2**	**3.9.E-04**	**4.3.E-02**	**Glucose tolerance**
**6**	**23**	**2**	**2.1.E-03**	**1.4.E-01**	**Triglycerides**
**7**	**42**	**2**	**6.8.E-03**	**2.3.E-01**	**Obesity**

The related annotations to adiposity is indicated in bold type.

### Case example 3: Network expansion + Cross-species GSA

Finally, the third case shows GSA with a combination of network expansion and cross-species GSA. As input, seven GWAS hit genes for venous thrombosis (VT) are used [26]. There is no significant GSA hit for phenotypic annotation using the simple cross-species GSA approach as in case 1 (3 input genes in mouse, VT_mouse). Apparently, the network expansion (43 input genes in human, Net_VT) resulted in some significant hits including *pregnancy loss* (q-value = 4.9e-9) and *brain hemorrhage* (q-value = 1.5e-4). However, the common genes between the input and the target gene set were only 2 ~ 4 genes due to the scarcity of human phenotypic annotation, making this GSA results less convincing (Table 5). Next, we created a network-expanded & orthology mapped set of 34 mouse genes (Net_VT_Mouse) by combining the features of both *set creator* and *network navigator*. It resulted in even richer phenotypic interpretations, having more than four times of GSA hits (45 annotations for Net_VT_Mouse shown in Table 6) than the human network-expanded set (11 annotations for Net_VT shown in Table 5) at the same cut-off (q-value < 0.05). These GSA hits include many vasculature-related diseases such as *abnormal blood coagulation* (q-value = 7.5e-17), *increased bleeding time* (q = -value = 7.0e-6), and *gastrointestinal hemorrhage* (q-value = 3.4e-5). It demonstrates that cross-species and network-expanded GSA allows even more sensitive and extensive interpretation of gene lists with improved statistical significance.

**Table 5:** The GSA result by a network-expanded GSA only (case example 3)

**Rank**	**Set size**	**Overlap**	**P-value**	**Q-value**	**Human phenotype**
1	5	4	9.3.E-12	4.9.E-09	Pregnancy loss, recurrent
2	12	4	9.1.E-10	2.4.E-07	Stroke
3	6	3	3.3.E-08	5.9.E-06	Stroke, ischemic
4	2	2	1.4.E-06	1.5.E-04	Fetal loss
**5**	**2**	**2**	**1.4.E-06**	**1.5.E-04**	**Brain hemorrhage**
6	4	2	8.6.E-06	7.6.E-04	Peripheral vascular disease
7	5	2	1.4.E-05	1.1.E-03	Polycystic ovary syndrome
8	7	2	3.0.E-05	2.0.E-03	Cerebrovascular disease
9	11	2	7.8.E-05	4.6.E-03	Myocardial infarction
10	12	2	9.3.E-05	5.0.E-03	Cardiovascular disease
11	25	2	4.2.E-04	2.0.E-02	Myocardial infarct

The related annotations to venous thrombosis is indicated in bold type.

**Table 6:** The GSA result by a network-expanded and cross-species GSA (case example 3)

**Rank**	**Set size**	**Overlap**	**P-value**	**Q-value**	**Mouse phenotype**
**1**	**79**	**10**	**9.9.E-21**	**7.5.E-17**	**Abnormal blood coagulation**
**2**	**73**	**5**	**1.9.E-09**	**7.0.E-06**	**Increased bleeding time**
**3**	**37**	**4**	**1.3.E-08**	**3.4.E-05**	**Gastrointestinal hemorrhage**
4	150	5	7.0.E-08	1.2.E-04	Increased leukocyte cell number
5	57	4	7.9.E-08	1.2.E-04	Abnormal cell adhesion
6	1258	9	1.5.E-07	1.9.E-04	Premature death
7	75	4	2.4.E-07	2.6.E-04	Decreased susceptibility to endotoxin shock
**8**	**80**	**4**	**3.1.E-07**	**2.9.E-04**	**Thrombosis**
9	94	4	6.0.E-07	5.0.E-04	Increased monocyte cell number
10	30	3	1.3.E-06	9.1.E-04	Abnormal cellular extravasation
**11**	**277**	**5**	**1.4.E-06**	**9.1.E-04**	**Hemorrhage**
**12**	**3**	**2**	**1.5.E-06**	**9.1.E-04**	**Purpura**
13	33	3	1.7.E-06	1.0.E-03	Decreased susceptibility to bacterial infection induced morbidity/mortality
14	153	4	4.1.E-06	2.1.E-03	Decreased erythrocyte cell number
15	153	4	4.1.E-06	2.1.E-03	Increased neutrophil cell number
16	50	3	6.1.E-06	2.9.E-03	Impaired macrophage chemotaxis
**17**	**51**	**3**	**6.5.E-06**	**2.9.E-03**	**Decreased platelet aggregation**
**18**	**6**	**2**	**7.2.E-06**	**3.0.E-03**	**Abnormal circulating fibrinogen level**
**19**	**7**	**2**	**1.0.E-05**	**3.8.E-03**	**Uterine hemorrhage**
**20**	**7**	**2**	**1.0.E-05**	**3.8.E-03**	**Petechiae**
21	60	3	1.1.E-05	3.8.E-03	Impaired neutrophil recruitment
22	8	2	1.4.E-05	4.4.E-03	Increased susceptibilityto infection induced morbidity/mortality
23	65	3	1.4.E-05	4.4.E-03	Abnormal leukocyte migration
24	211	4	1.5.E-05	4.6.E-03	Increased sensitivityto induced morbidity/mortality
25	69	3	1.6.E-05	4.8.E-03	Increased eosinophil cell number
26	70	3	1.7.E-05	4.8.E-03	Abnormal myelopoiesis
27	9	2	1.7.E-05	4.8.E-03	Hemothorax
28	72	3	1.8.E-05	4.9.E-03	Abnormal cell migration
**29**	**81**	**3**	**2.6.E-05**	**6.4.E-03**	**Internal hemorrhage**
**30**	**11**	**2**	**2.6.E-05**	**6.4.E-03**	**Skin hemorrhage**
**31**	**11**	**2**	**2.6.E-05**	**6.4.E-03**	**Abnormal platelet aggregation**
32	86	3	3.1.E-05	7.3.E-03	Decreased angiogenesis
33	13	2	3.8.E-05	8.5.E-03	Decreased susceptibility to induced choroidal neovascularization
34	100	3	4.9.E-05	1.1.E-02	Abnormal inflammatory response
35	289	4	4.9.E-05	1.1.E-02	Anemia
**36**	**15**	**2**	**5.0.E-05**	**1.1.E-02**	**Pulmonary alveolar hemorrhage**
37	18	2	7.3.E-05	1.5.E-02	Abnormal uterine environment
38	20	2	9.1.E-05	1.8.E-02	Abnormal physiological neovascularization
**39**	**21**	**2**	**1.0.E-04**	**1.9.E-02**	**Hemoperitoneum**
40	22	2	1.1.E-04	2.0.E-02	Pregnancy-related premature death
**41**	**22**	**2**	**1.1.E-04**	**2.0.E-02**	**Abnormal platelet activation**
**42**	**133**	**3**	**1.1.E-04**	**2.0.E-02**	**Decreased platelet cell number**
43	24	2	1.3.E-04	2.3.E-02	Increased susceptibility to fungal infection
44	33	2	2.5.E-04	4.3.E-02	Decreased cerebral infarction size
45	180	3	2.7.E-04	4.6.E-02	Pallor

The related annotations to venous thrombosis is indicated in bold type.

## Conclusion

*gsGator* is a fully integrated, web-based tool for gene set analysis (GSA), which allows highly flexible and interactive GSA analyses. A series of new gene sets can be created as a combination of any existing gene sets, which is highly desirable in most exploratory and discovery-oriented studies. According to our survey, cross-species GSA expands the coverage of phenotypic annotation by 5.4% and PPI network by 13.3% for human genes, respectively. Few existing tools are equipped with these functionalities in a single unified database, reducing the burden of consulting multiple web sites and bioinformatics tools. All the gene lists and analytic results can be exported for further processing and integration with other analytic results. As demonstrated in the three case examples, interactive and cross-species GSA greatly extends the scope and utility of GSA, leading to novel insights via conserved functional gene modules across different species.

## Availability and requirements

gsGator is freely available at http://gsGator.ewha.ac.kr. gsGator user interface is implemented using Adobe FLEX 5.0 and the internal system is operated using JAVA and Apache web server. MySQL 5.5 is used for database management. Flash player and JAVA need be installed for using gsGator. Currently, gsGator is optimized and tested for Chrome or Firefox web browser in both Linux and Windows environment. Some of the features may be limited for other types of web browser e.g. Internet Explorer.

## Competing interests

The authors declare that they have no competing interests.

## Authors’ contributions

HK, SL and WK participated in the design of the study, conducted computational and statistical analysis and wrote the manuscript. SC and DR participated in collecting data sets and build databases. IC implemented the web application and computational analysis. All authors read and approved the final manuscript.

## Supplementary Material

Additional file 1: Table S1 Supported gene ID types in gsGator. Click here for file

Additional file 2 The usage examples of simple GSA, cross-species GSA, and/or network-expanded GSA. Click here for file
