# Supplementary material for: gsGator: an integrated web platform for cross-species gene set analysis
Source: BMC Bioinformatics. 2014 Jan 14;15:13. doi: 10.1186/1471-2105-15-13 (PMC3898093; doi:10.1186/1471-2105-15-13)
Supplement: Additional file 1: Table S1 — Supported gene ID types in gsGator. [file 1471-2105-15-13-S1.pdf]

Table S1. Supported gene ID types in gsGator

Category	ID Type	Example
**Gene ID**	Entrez Gene ID	1,2
	Symbol	A1BG, A2M
	Synonym	HYST2477, CPAMD5
	HGNC Symbol	A1BG, A2M
	HGNC ID	5,7
	RefSeq ID	NM_130786, NM_000014, NP_000005
	GenBank ID	224514627, 224514867
	GenBank Accession	AK055885, CR749334
	UniGene	Hs.529161, Hs.212838
	Ensembl Gene ID	ENSG00000121410, ENSG00000175899
	Ensembl Transcript ID	ENST00000263100, ENST00000318602
	Vega	OTTHUMG00000150267
	Affymetrix Probe ID	8039750, 11741180_a_at, RC_H94666_at
	Agilent Probe ID	A_23_P38816, A_23_P116898
	Illumina Probe ID	cg03630821, ILMN_1745607
	MGI	MGI:87854, MGI:1328365
	FlyBase	FBgn0195790, FBgn0195792
	EcoCyc	G7918, EG11740
	TAIR	AT3G30630, AT3G30620
	SGD	S000000580, S000000579
	WormPep ID	CE11958, CE23980
	WormBase Transcript ID	K08F11.5.2, T13F2.3b
	WormBaseSeq Name	WBGene00019544, WBGene00004031
**Protein ID**	Ensembl Protein ID	ENSP00000263100, ENSP00000323929
	UniProtKB ID	A1BG_HUMAN, A2MG_HUMAN
	UniProtKB Accession	P04217, P01023
