# Supplementary material for: gsGator: an integrated web platform for cross-species gene set analysis
Source: BMC Bioinformatics. 2014 Jan 14;15:13. doi: 10.1186/1471-2105-15-13 (PMC3898093; doi:10.1186/1471-2105-15-13)
Supplement: Additional file 2 — The usage examples of simple GSA, cross-species GSA, and/or network-expanded GSA. [file 1471-2105-15-13-S2.pdf]

Case I> Phenotypic Interpretation of GWAS results for eye color

OPEN ACCESS Freely available online PLoS GENETICS

Web-Based, Participant-Driven Studies Yield Novel Genetic Associations for Common Traits

Nicholas Eriksson[1]*, J. Michael Macpherson[1], Joyce Y. Tung[1], Lawrence S. Hon[1], Brian Naughton[1], Serge Saxonov[1], Linda Avey[1], Anne Wojcicki[1], Itsik Pe'er[2], Joanna Mountain[1,3]*

1 23andMe, Mountain View, California, United States of America, **2** Department of Computer Science, Columbia University, New York, New York, United States of America, **3** Department of Anthropology, Stanford University, Stanford, California, United States of America

We took a GWAS case for eye color phenotype (Eriksson et al. 2010), where 5 genes are reported as significantly associated. Using the *Set Creator*, a gene set consisting of these 5 human genes was named as **EC_Eriksson**. An orthologous set for mouse, **EC_Eriksson_Mouse** is also created as an orthologous gene set, where 4 out of 5 genes were mapped by orthology.

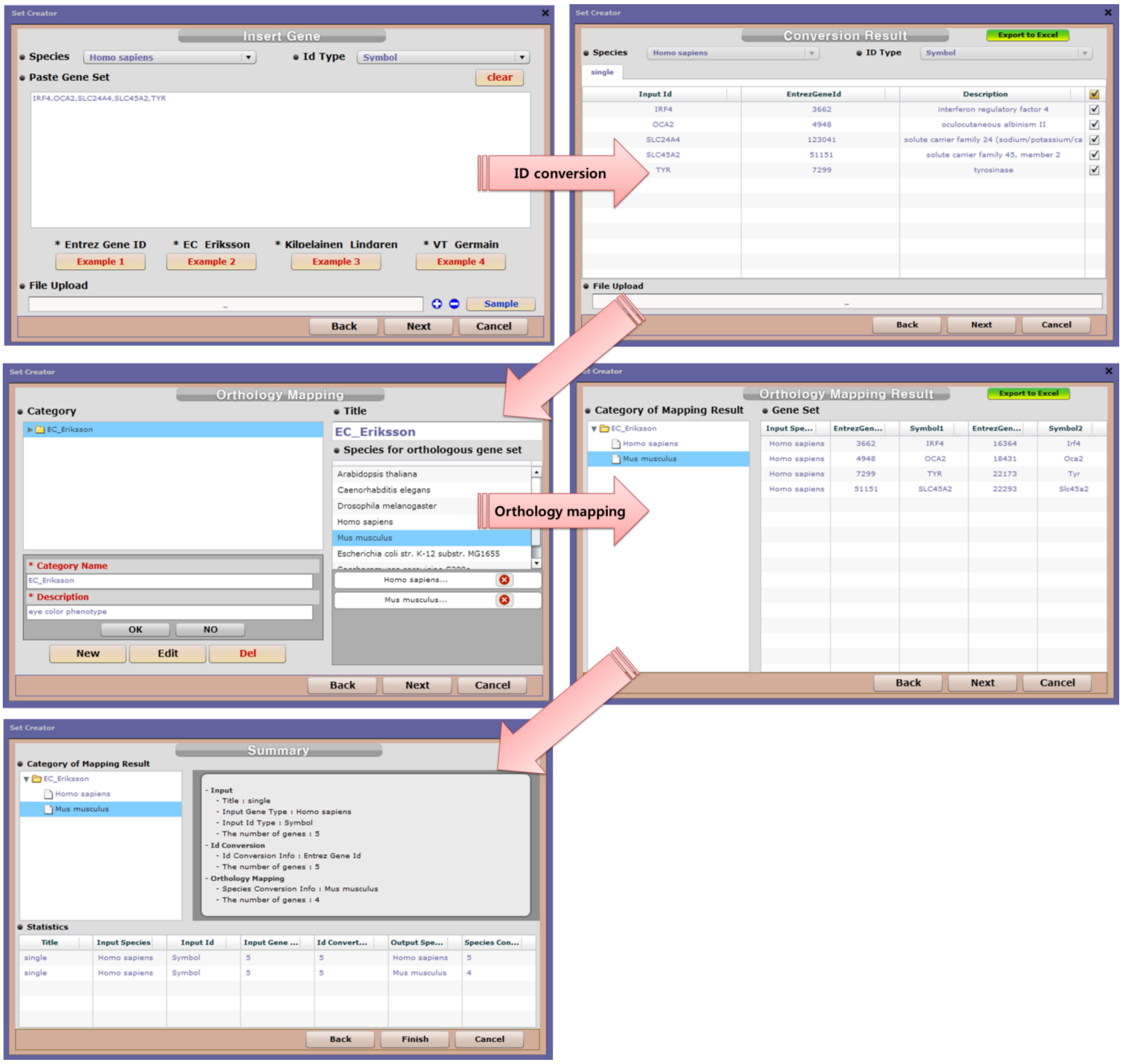

EC_Eriksson		EC_Eriksson_Mouse	
**Symbol**	**Entrez Gene ID**	**Symbol**	**Entrez Gene ID**
IRF4	3662	Irf4	16364
OCA2	4948	Oca2	18431
SLC45A2	51151	Slc45a2	22293
TYR	7299	Tyr	22173
SLC24A4	123041	-	-

GSA of **EC_Eriksson** for human phenotypic annotations (GAD and OMIM) gave no result because they mostly consist of single gene annotation (gene set size = 1), lacking statistical significance. (In gsGator, GSA threshold is set as $|A \cap B| > 1$). However, the GSA with **EC_Eriksson_Mouse** resulted in many hits for phenotypic annotations on eye/skin pigmentation and coat color. As shown in this example, model organisms are a rich source of phenotypic information, which also is growing more rapidly than in human.

The cross-species GSA result against mouse phenotype annotations in gsGator

Rank by Q-value	Set Size	Overlap Size	P-value	Q-value	Kappa	Mouse Phenotypic Annotations
1	22	3	3.31.E-10	2.49.E-06	2.31.E-01	decreased eye pigmentation
2	30	3	8.74.E-10	3.28.E-06	1.76.E-01	abnormal eye pigmentation
3	51	3	4.48.E-09	1.12.E-05	1.09.E-01	abnormal skin pigmentation
4	76	3	1.51.E-08	2.84.E-05	7.49.E-02	diluted coat color
5	87	3	2.28.E-08	3.43.E-05	6.58.E-02	abnormal coat/hair pigmentation
6	5	2	5.18.E-08	5.56.E-05	4.44.E-01	absent eye pigmentation
7	5	2	5.18.E-08	5.56.E-05	4.44.E-01	ocular albinism
8	7	2	1.09.E-07	1.02.E-04	3.64.E-01	variegated coat color
9	8	2	1.45.E-07	1.09.E-04	3.33.E-01	abnormal choroid melanin granule morphology
10	8	2	1.45.E-07	1.09.E-04	3.33.E-01	mottled coat
11	9	2	1.86.E-07	1.27.E-04	3.08.E-01	abnormal melanogenesis
12	18	2	7.92.E-07	4.96.E-04	1.82.E-01	yellow coat color
13	24	2	1.43.E-06	8.26.E-04	1.43.E-01	abnormal melanosome morphology
14	27	2	1.82.E-06	9.76.E-04	1.29.E-01	irregular coat pigmentation
15	31	2	2.41.E-06	1.21.E-03	1.14.E-01	hypopigmentation
16	87	2	1.93.E-05	9.08.E-03	4.38.E-02	prenatal lethality
17	975	3	3.25.E-05	1.44.E-02	5.96.E-03	decreased body size
18	125	2	3.99.E-05	1.67.E-02	3.09.E-02	infertility
19	237	2	1.43.E-04	5.39.E-02	1.64.E-02	increased susceptibility to bacterial infection
20	365	2	3.39.E-04	9.10.E-02	1.07.E-02	complete prenatal lethality
21	519	2	6.82.E-04	1.11.E-01	7.48.E-03	male infertility
22	574	2	8.32.E-04	1.25.E-01	6.76.E-03	complete postnatal lethality
23	1258	2	3.89.E-03	2.90.E-01	3.00.E-03	premature death
24	1642	2	6.51.E-03	3.92.E-01	2.26.E-03	no abnormal phenotype detected

In the next step, the GSA results were visualized using ***network viewer*** by clicking 'show' button at the result page. It shows the network among the

gene sets, where the input gene set (**EC_Eriksson_Mouse**) is located at the center and the GSA hit annotations are located peripherally.

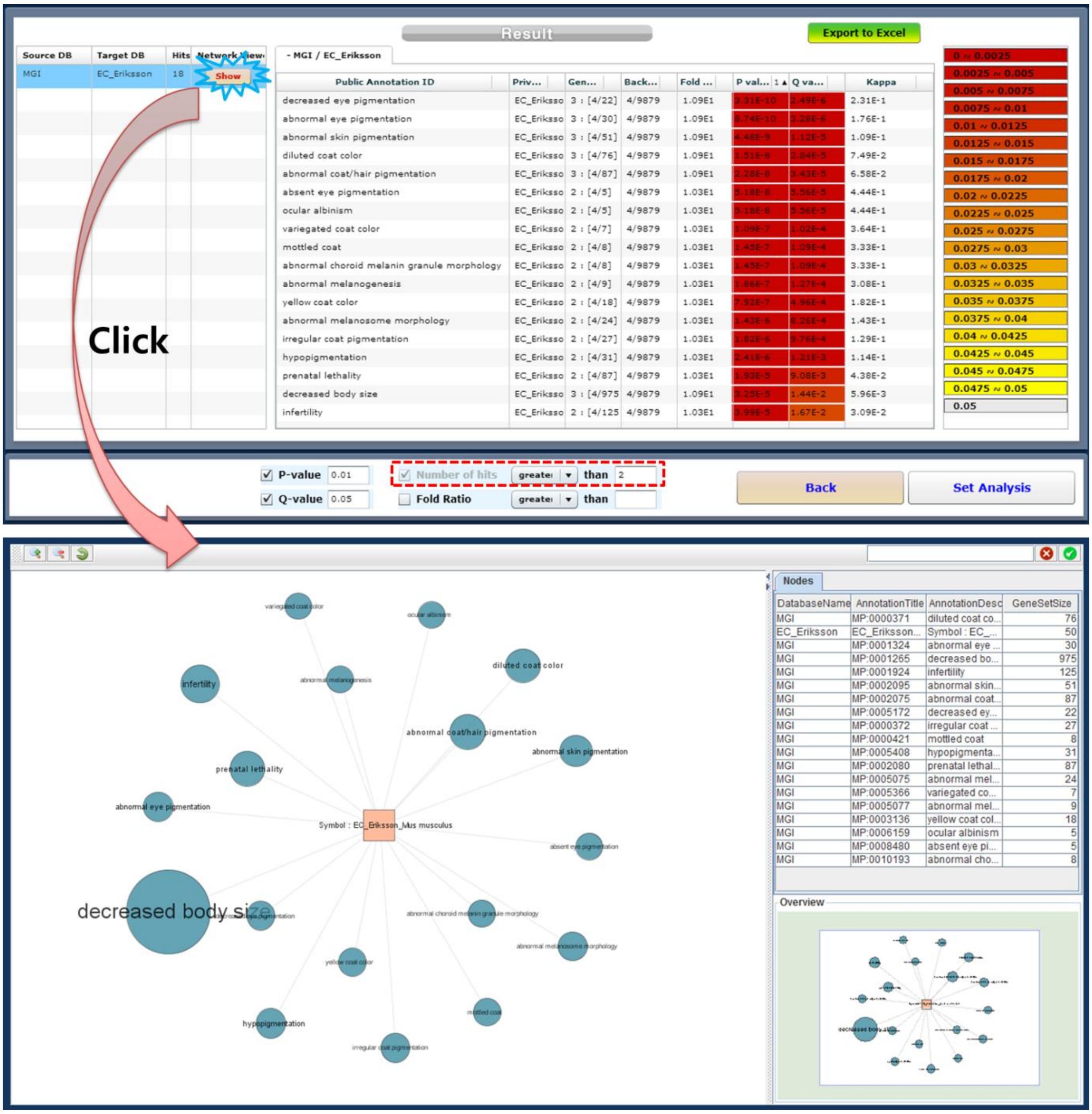

Case II> Network-expanded GSA for more sensitive interpretation of adiposity-associated genes from GWAS.

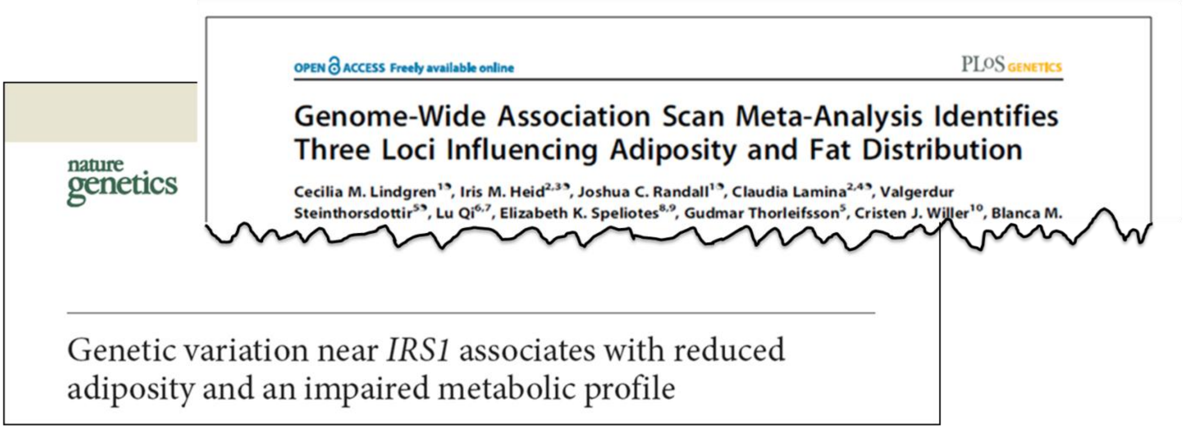
OPEN ACCESS Freely available online

PLoS GENETICS

Genome-Wide Association Scan Meta-Analysis Identifies Three Loci Influencing Adiposity and Fat Distribution

Cecilia M. Lindgren[1¶], Iris M. Heid[2,3¶], Joshua C. Randall[1¶], Claudia Lamina[2,4¶], Valgerdur Steinthorsdottir[5¶], Lu Qi[6,7], Elizabeth K. Speliotes[8,9], Gudmar Thorleifsson[5], Cristen J. Willer[10], Blanca M.

nature genetics

Genetic variation near *IRS1* associates with reduced adiposity and an impaired metabolic profile

Here, we took the two GWAS cases for adiposity (Kilpelainen et al. 2011 and Lindgren et al. 2009), which causes obesity and diabetes. The union of the associated genes near the susceptibility loci is defined as **Kilpelainen_Lindgren** using *Set Creator*. Further, we also created an extended gene set by including network neighbor genes in PPI (protein-protein interaction network) using *Network Navigator*. User may expand gene set using other types of network such as TF-target and miRNA-target.

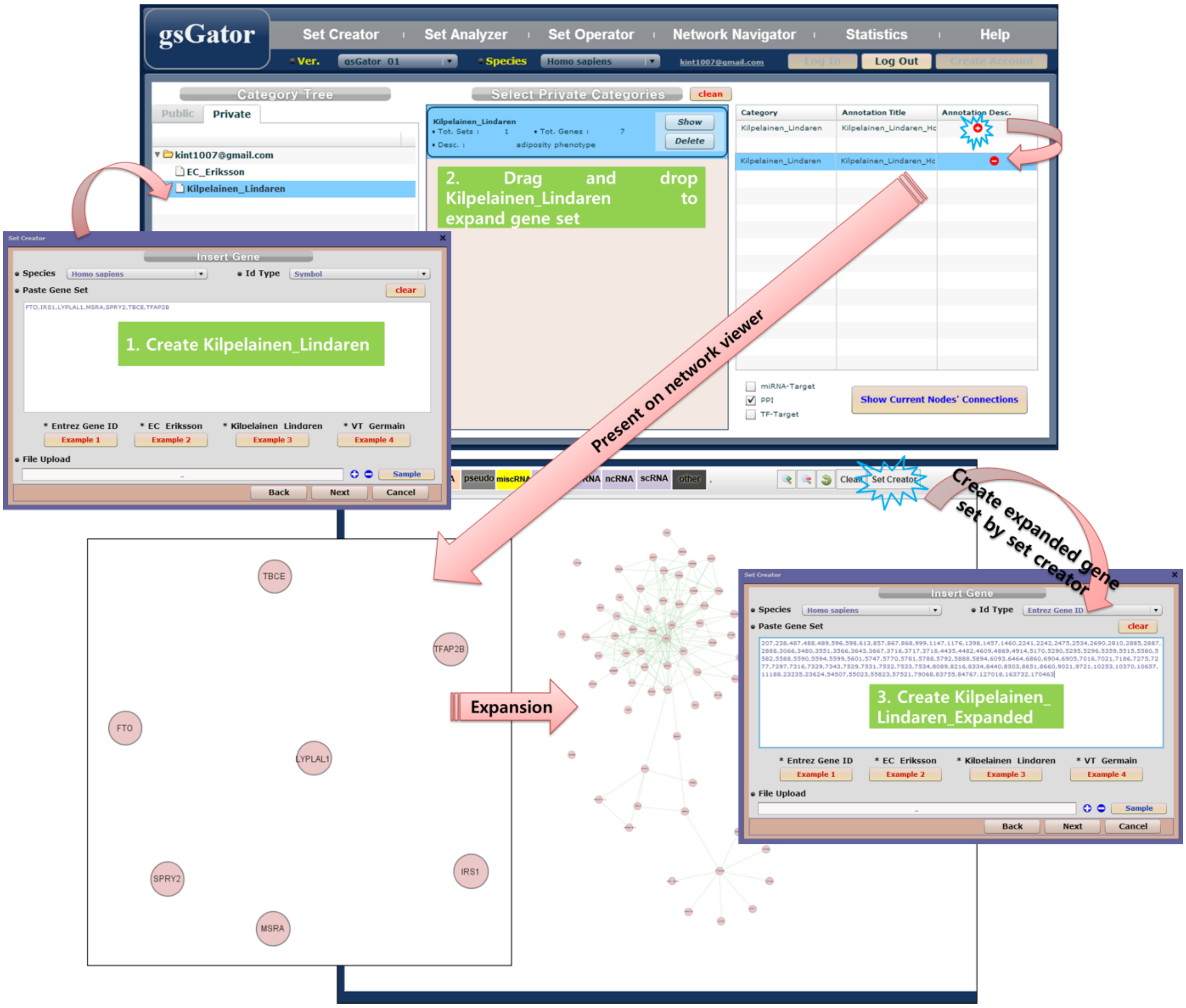

Kilpelainen_Lindgren_Expanded									
Kilpelainen_Lindgren		Network Neighbor Genes (PPI)							
Symbol	Entrez Gene ID	Symbol	Entrez Gene ID	Symbol	Entrez Gene ID	Symbol	Entrez Gene ID	Symbol	Entrez Gene ID
FTO	79068	ADAMTSL4	54507	GHR	2690	NPM1	4869	SIK2	23235
IRS1	3667	AKT1	207	GPRIN2	9721	NTRK1	4914	SOCS1	8651
LYPLAL1	127018	ALK	238	GRB10	2887	PDPK1	5170	SOCS3	9021
MSRA	4482	AP3S1	1176	GRB14	2888	PHIP	55023	SSBP4	170463
SPRY2	10253	ATP2A1	487	GRB2	2885	PIK3CA	5290	SYT4	6860
TBCE	6905	ATP2A2	488	HDAC2	3066	PIK3R1	5295	TBCD	6904
TFAP2B	7021	ATP2A3	489	HIST1H2AC	8334	PIK3R2	5296	TESK1	7016
		BCL2	596	IGF1R	3480	PIK3R3	8503	TRAF2	7186
		BCL2L1	598	IKBKB	3551	PLSCR1	5359	TRIM51	84767
		BCR	613	IL4R	3566	PPP2CA	5515	TUB	7275
		CAV1	857	INSR	3643	PRKCD	5580	TUBA4A	7277
		CBL	867	IRS2	8660	PRKCG	5582	TYK2	7297
		CBLB	868	JAK1	3716	PRKCQ	5588	UBC	7316
		CBLC	23624	JAK2	3717	PRKCZ	5590	UBE2I	7329
		CDH1	999	JAK3	3718	PTK2	5747	UBTF	7343
		CHUK	1147	KHDRBS1	10657	PTPN1	5770	VPS11	55823
		CITED1	4435	KRTAP4-12	83755	PTPN11	5781	YEATS4	8089
		CITED2	10370	LZTR1	8216	PTPRC	5788	YWHAB	7529
		CITED4	163732	MAPK1	5594	PTPRF	5792	YWHAE	7531
		CRK	1398	MAPK8	5599	RAD51	5888	YWHAG	7532
		CSNK2A1	1457	MAPK9	5601	RAF1	5894	YWHAH	7533
		CSNK2B	1460	MTOR	2475	ROCK1	6093	YWHAZ	7534
		FER	2241	MYC	4609	RPTOR	57521		
		FES	2242	NCK2	8440	SFN	2810		
		FYN	2534	NISCH	11188	SHC1	6464		

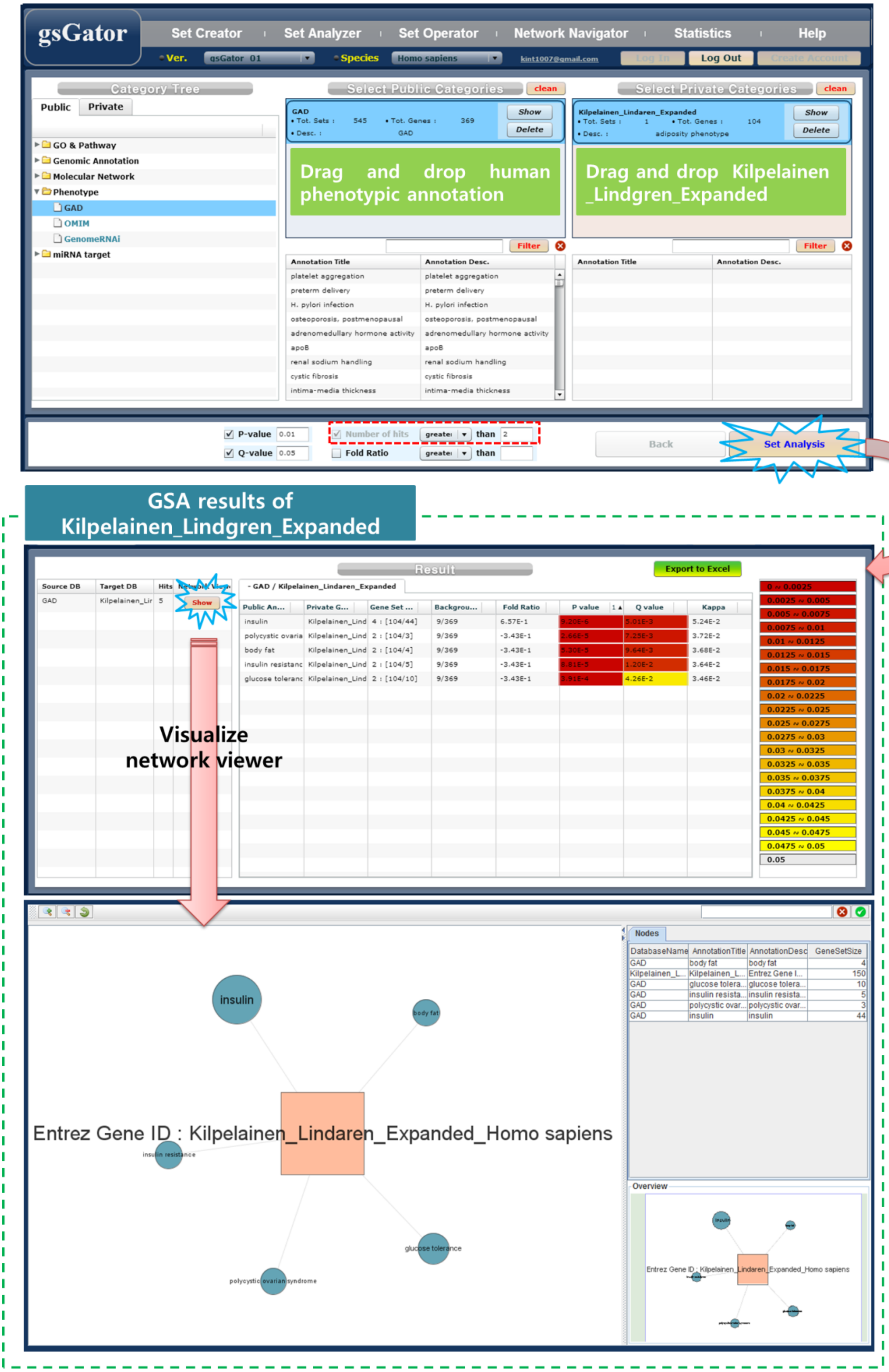

gsGator
Set Creator
Set Analyzer
Set Operator
Network Navigator
Statistics
Help
Log Out
Category Tree
Select Public Categories
Select Private Categories
Drag and drop human phenotypic annotation
Drag and drop Kilpelainen _Lindgren_Expanded
Set Analysis
GSA results of Kilpelainen_Lindgren_Expanded
Result
Export to Excel
Visualize network viewer
Entrez Gene ID : Kilpelainen_Lindaren_Expanded_Homo sapiens
insulin
body fat
insulin resistance
glucose tolerance
polycystic ovarian syndrome

While the original input gene set (**Kilpelainen_Lindgren**) resulted in no significant hits when GSA was performed against human phenotypic annotations (GAD and OMIM category)**,** the network-expanded set (**Kilpelainen_Lindgren_Expanded**) resulted in multiple GSA hits closely related to adiposity, such as body fat, insulin/glucose metabolism and obesity. It shows that network-expanded GSA can identify related phenotypic annotations more sensitively by increasing its statistical power.

Network_expanded GSA result against human phenotype annotations in gsGator (GAD and OMIM).

Rank by Q-value	Set Size	Overlap Size	P-value	Q-value	Kappa	Human Phenotypic Annotations
1	44	4	9.20196E-06	0.00501507	0.05236555	insulin
2	3	2	2.66037E-05	0.00724951	0.03722139	polycystic ovarian syndrome
3	4	2	5.3051E-05	0.0096376	0.03682323	body fat
4	5	2	8.81584E-05	0.01201158	0.03643234	insulin resistance
5	10	2	0.000390916	0.04260985	0.03458016	glucose tolerance
6	23	2	0.002115271	0.13609951	0.03044369	triglycerides
7	42	2	0.00680673	0.23185425	0.02571748	obesity

Case III> Combination of network-expansion & cross-species GSA for interpreting GWAS result for venous thrombosis

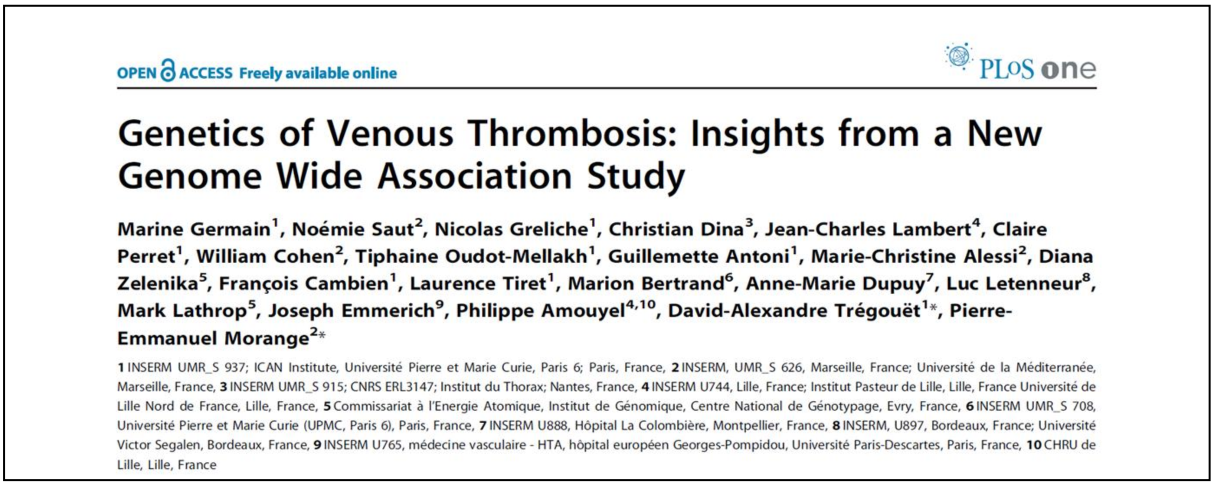
OPEN ACCESS Freely available online — PLoS one

Genetics of Venous Thrombosis: Insights from a New Genome Wide Association Study

Marine Germain[1], Noémie Saut[2], Nicolas Greliche[1], Christian Dina[3], Jean-Charles Lambert[4], Claire Perret[1], William Cohen[2], Tiphaine Oudot-Mellakh[1], Guillemette Antoni[1], Marie-Christine Alessi[2], Diana Zelenika[5], François Cambien[1], Laurence Tiret[1], Marion Bertrand[6], Anne-Marie Dupuy[7], Luc Letenneur[8], Mark Lathrop[5], Joseph Emmerich[9], Philippe Amouyel[4,10], David-Alexandre Trégouët[1]*, Pierre-Emmanuel Morange[2]*

1 INSERM UMR_S 937; ICAN Institute, Université Pierre et Marie Curie, Paris 6; Paris, France, **2** INSERM, UMR_S 626, Marseille, France; Université de la Méditerranée, Marseille, France, **3** INSERM UMR_S 915; CNRS ERL3147; Institut du Thorax; Nantes, France, **4** INSERM U744, Lille, France; Institut Pasteur de Lille, Lille, France Université de Lille Nord de France, Lille, France, **5** Commissariat à l'Energie Atomique, Institut de Génomique, Centre National de Génotypage, Evry, France, **6** INSERM UMR_S 708, Université Pierre et Marie Curie (UPMC, Paris 6), Paris, France, **7** INSERM U888, Hôpital La Colombière, Montpellier, France, **8** INSERM, U897, Bordeaux, France; Université Victor Segalen, Bordeaux, France, **9** INSERM U765, médecine vasculaire - HTA, hôpital européen Georges-Pompidou, Université Paris-Descartes, Paris, France, **10** CHRU de Lille, Lille, France

We took a GWAS case for venous thrombosis (VT) (Germain et al. 2011), which is blood coagulation in vein. The list of genes at the susceptibility loci is defined as **VT_Germain**, which consists of 7 genes. Out of the 7 genes, only 3 were mapped to mouse genes by orthology, which were defined as **VT_Mouse**. We further created **Net_VT** (network-expanded) and **Net_VT_Mouse** (network-expanded & mouse ortholog mapped) using *Network Navigator* and *Set Creator* similarly in the above examples. The set sizes of **Net_VT** and **Net_VT_Mouse** increased to 42 genes (7 input genes + 35 network-expanded) and 34 genes (3 input orthologs + 31 network-expanded orthologs) respectively.

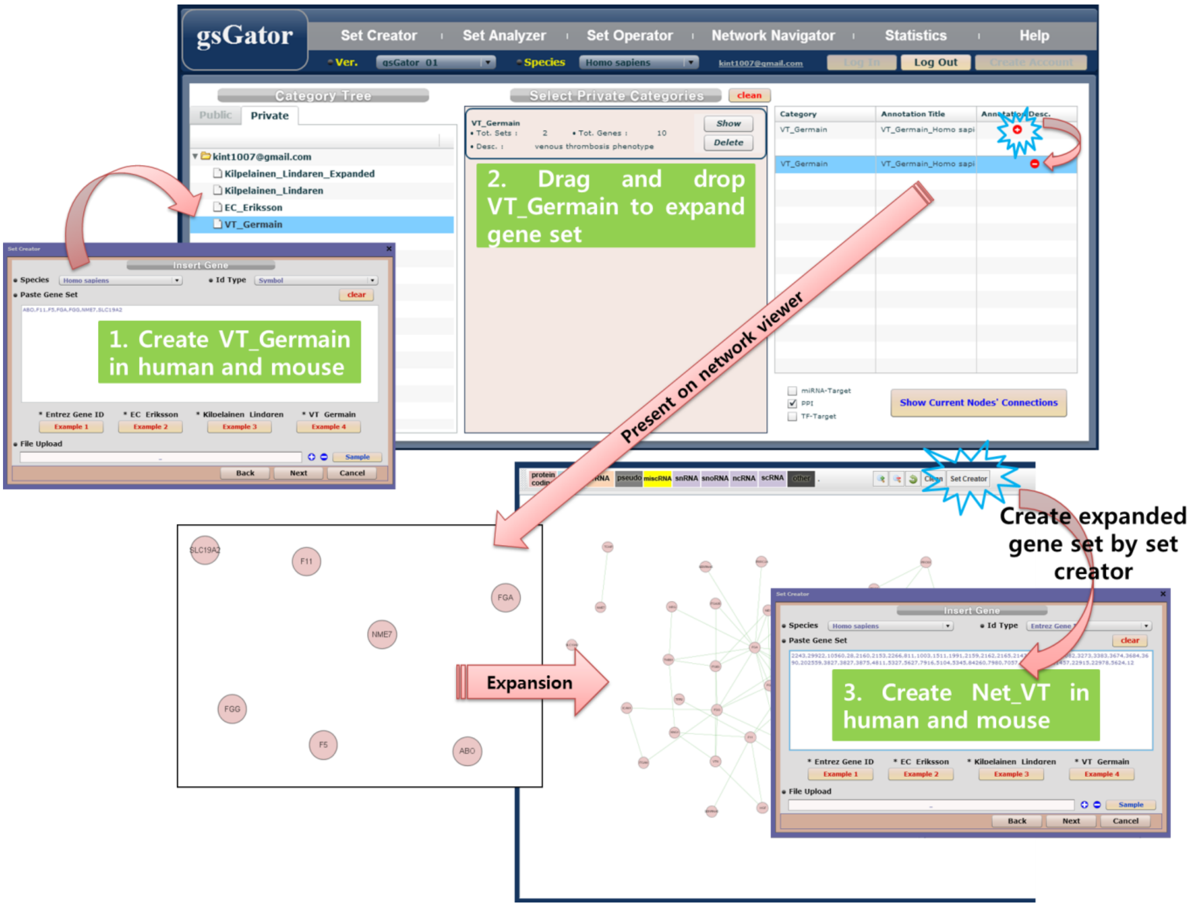

VT | VT_Mouse

Human Symbol	Human Entrez Gene ID	Mouse Symbol	Mouse Entrez Gene ID	Human Symbol	Human Entrez Gene ID	Mouse Symbol	Mouse Entrez Gene ID
FGA	2243	Fga	14161	F11	2160		
NME7	29922	Nme7	171567	F5	2153		
SLC19A2	10560	Slc19a2	116914	FGG	2266		
ABO	28						
CALR	811	Calr	12317	KNG1	3827	Kng2	385643
CDH5	1003	Cdh5	12562	KRT18	3875	Krt18	16668
CTSG	1511	Ctsg	13035	NID1	4811	Nid1	18073
ELANE	1991	Elane	50701	PLAT	5327	Plat	18791
F10	2159	F10	14058	PROS1	5627	Pros1	19128
F13A1	2162	F13a1	74145	PRRC2A	7916	Prrc2a	53761
F13B	2165	F13b	14060	SERPINA5	5104	Serpina5	268591
F2	2147	F2	14061	SERPINF2	5345	Serpinf2	18816
FGB	2244	Fgb	110135	TCHP	84260	Tchp	77832
FKBP6	8468	Fkbp6	94244	TFPI2	7980	Tfpi2	21789
HGF	3082	Hgf	15234	THBS1	7057	Thbs1	21825
HRG	3273	Hrg	94175	VTN	7448	Vtn	22370
ICAM1	3383	Icam1	15894	ZNF263	10127	Zfp263	74120
ITGA2B	3674	Itga2b	16399	CSNK2A1	1457		
ITGAM	3684	Itgam	16409	MMRN1	22915		
ITGB3	3690	Itgb3	16416	NT5C2	22978		
KHDRBS2	202559	Khdrbs2	170771	PROC	5624		
KNG1	3827	Kng1	16644	SERPINA3	12		

Net_VT | Net_VT_Mouse

GSA using the input gene set (**VT**) had no phenotypic hits (GAD, OMIM). The mouse orthologous set (**VT_Mouse**) has only 3 genes and GSA shows only a single significant hit (**MGI_ID**: premature death in MGI, Q-value > 0.9), which is not very informative.

The GSA result for the mouse orthologous set (VT_Mouse)

Set Size	Overlap Size	P-value	Q-value	Kappa	Mouse Phenotypic Annotations
1258	2	0.001995	0.911464	0.003048	premature death

Apparently, the network expansion (43 input genes in human, **Net_VT**) resulted in some significant hits including pregnancy loss (q-value=4.9e-9) and brain hemorrhage (q-value=1.5e-4). However, the common genes between the input and the target gene set were only 2~4 genes due to the scarcity of human phenotypic annotation, making this GSA results less convincing. However, even more phenotypic annotations are listed for the network-expanded & orthology-mapped mouse set (**Net_VT_Mouse**), which are directly related to thrombosis and statistically more significant. At the same cut-off of q-value < 0.05, Net_VT_Mouse resulted in more than four times of GSA hits than Net_VT. It demonstrates that cross-species and network-expanded GSA allows even more sensitive and extensive interpretation of gene lists with improved statistical significance.

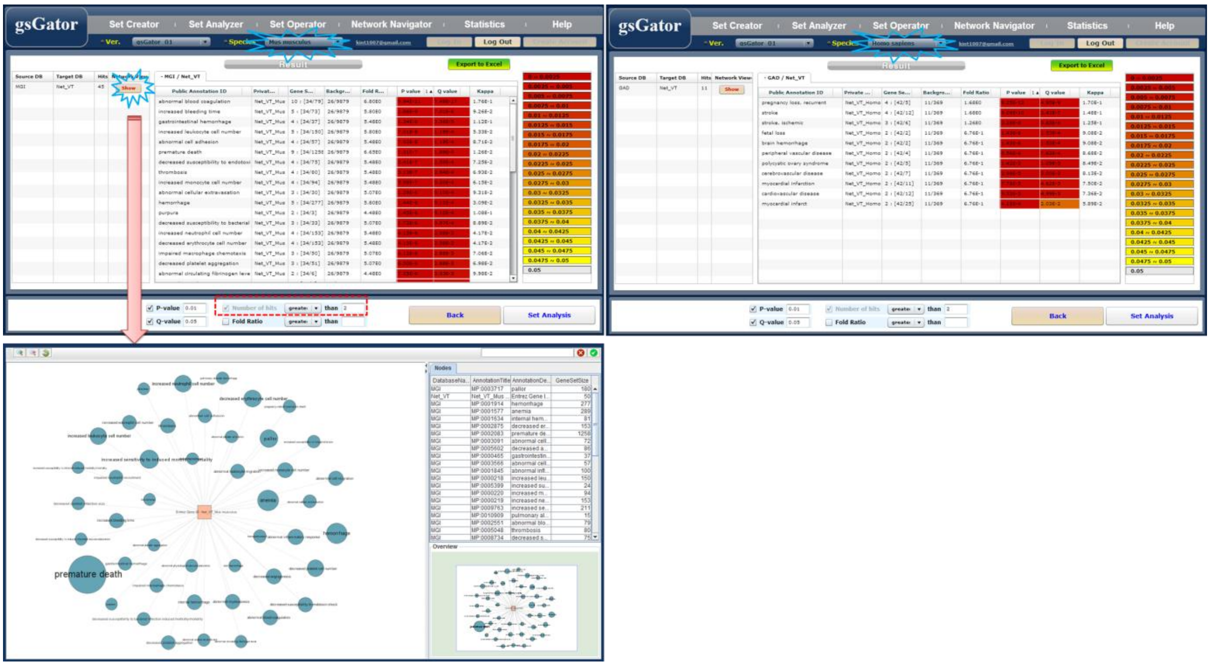
gsGator
Set Creator
Set Analyzer
Set Operator
Network Navigator
Statistics
Help
Log Out
Export to Excel
Back
Set Analysis
premature death

The GSA hits for the network-expanded, human gene set (Net_VT)						
**Rank by Q-value**	**Set Size**	**Overlap Size**	**P-value**	**Q-value**	**Kappa**	**Human Phenotypic Annotations**
1	5	4	9.25036E-12	4.94651E-09	0.16999905	pregnancy loss, recurrent
2	12	4	9.08788E-10	2.42981E-07	0.14768972	stroke
3	6	3	3.28488E-08	5.85515E-06	0.12473519	stroke, ischemic
4	2	2	1.42975E-06	0.000152909	0.09080906	fetal loss
5	2	2	1.42975E-06	0.000152909	0.09080906	brain hemorrhage
6	4	2	8.55876E-06	0.000762781	0.08676431	peripheral vascular disease
7	5	2	1.42482E-05	0.001088431	0.08487075	polycystic ovary syndrome
8	7	2	2.98522E-05	0.001995384	0.081315	cerebrovascular disease
9	11	2	7.78244E-05	0.004623954	0.07500703	myocardial infarction
10	12	2	9.32816E-05	0.004988112	0.07357578	cardiovascular disease
11	25	2	0.000417695	0.020305164	0.05885151	myocardial infarct

Some of the GSA hits for the network-expanded & orthology-mapped mouse gene set (NET_VT_Mouse)						
Rank by Q-value	Set Size	Overlap (|A ∩ B|)	P-value	Q-value	Kappa	Mouse Phenotypic Annotations
1	79	10	9.94.E-21	7.48.E-17	1.76.E-01	abnormal blood coagulation
2	73	5	1.86.E-09	7.01.E-06	9.26.E-02	increased bleeding time
3	37	4	1.34.E-08	3.36.E-05	1.12.E-01	gastrointestinal hemorrhage
4	150	5	7.01.E-08	1.19.E-04	5.33.E-02	increased leukocyte cell number
5	57	4	7.93.E-08	1.19.E-04	8.71.E-02	abnormal cell adhesion
6	1258	9	1.51.E-07	1.89.E-04	1.26.E-02	premature death
7	75	4	2.41.E-07	2.59.E-04	7.25.E-02	decreased susceptibility to endotoxin shock
8	80	4	3.13.E-07	2.94.E-04	6.93.E-02	thrombosis
9	94	4	5.98.E-07	5.00.E-04	6.15.E-02	increased monocyte cell number
10	30	3	1.29.E-06	9.10.E-04	9.31.E-02	abnormal cellular extravasation
11	277	5	1.44.E-06	9.10.E-04	3.09.E-02	hemorrhage
12	3	2	1.45.E-06	9.10.E-04	1.08.E-01	purpura
13	33	3	1.72.E-06	9.97.E-04	8.89.E-02	decreased susceptibility to bacterial infection induced morbidity/mortality
14	153	4	4.15.E-06	2.08.E-03	4.17.E-02	decreased erythrocyte cell number
15	153	4	4.15.E-06	2.08.E-03	4.17.E-02	increased neutrophil cell number
16	50	3	6.12.E-06	2.88.E-03	7.06.E-02	impaired macrophage chemotaxis
17	51	3	6.50.E-06	2.88.E-03	6.98.E-02	decreased platelet aggregation
18	6	2	7.25.E-06	3.03.E-03	9.98.E-02	abnormal circulating fibrinogen level
19	7	2	1.01.E-05	3.80.E-03	9.73.E-02	uterine hemorrhage
20	7	2	1.01.E-05	3.80.E-03	9.73.E-02	petechiae
21	60	3	1.06.E-05	3.80.E-03	6.30.E-02	impaired neutrophil recruitment
22	8	2	1.35.E-05	4.42.E-03	9.50.E-02	increased susceptibility to infection induced morbidity/mortality
23	65	3	1.35.E-05	4.42.E-03	5.97.E-02	abnormal leukocyte migration
24	211	4	1.46.E-05	4.58.E-03	3.15.E-02	increased sensitivity to induced morbidity/mortality
25	69	3	1.62.E-05	4.83.E-03	5.74.E-02	increased eosinophil cell number
26	70	3	1.69.E-05	4.83.E-03	5.68.E-02	abnormal myelopoiesis
27	9	2	1.74.E-05	4.83.E-03	9.28.E-02	hemothorax
28	72	3	1.84.E-05	4.93.E-03	5.57.E-02	abnormal cell migration
29	81	3	2.61.E-05	6.42.E-03	5.12.E-02	internal hemorrhage
30	11	2	2.65.E-05	6.42.E-03	8.86.E-02	skin hemorrhage
31	11	2	2.65.E-05	6.42.E-03	8.86.E-02	abnormal platelet aggregation
32	86	3	3.12.E-05	7.34.E-03	4.90.E-02	decreased angiogenesis
33	13	2	3.75.E-05	8.55.E-03	8.47.E-02	decreased susceptibility to induced choroidal neovascularization
34	100	3	4.89.E-05	1.05.E-02	4.38.E-02	abnormal inflammatory response
35	289	4	4.94.E-05	1.05.E-02	2.35.E-02	anemia
36	15	2	5.04.E-05	1.05.E-02	8.12.E-02	pulmonary alveolar hemorrhage
37	18	2	7.33.E-05	1.49.E-02	7.65.E-02	abnormal uterine environment
38	20	2	9.09.E-05	1.80.E-02	7.36.E-02	abnormal physiological neovascularization
39	21	2	1.00.E-04	1.94.E-02	7.22.E-02	hemoperitoneum
40	22	2	1.10.E-04	2.03.E-02	7.09.E-02	pregnancy-related premature death
41	22	2	1.10.E-04	2.03.E-02	7.09.E-02	abnormal platelet activation
42	133	3	1.14.E-04	2.03.E-02	3.48.E-02	decreased platelet cell number
43	24	2	1.32.E-04	2.30.E-02	6.84.E-02	increased susceptibility to fungal infection
44	33	2	2.51.E-04	4.28.E-02	5.90.E-02	decreased cerebral infarction size
45	180	3	2.75.E-04	4.59.E-02	2.69.E-02	pallor
